# Income Inequality, Economic Growth and Stroke Mortality in Brazil: Longitudinal and Regional Analysis 2002-2009

**DOI:** 10.1371/journal.pone.0137332

**Published:** 2015-09-09

**Authors:** Natalia Vincens, Martin Stafström

**Affiliations:** 1 Social Medicine and Global Health, Department of Clinical Sciences Malmö, Lund University—Malmö, Malmö, Sweden; 2 CAPES Foundation, Ministry of Education of Brazil, Brasilia—DF, Brazil; Hunter College, UNITED STATES

## Abstract

**Background and Purpose:**

Stroke accounts for more than 10% of all deaths globally and most of it occurs in low- and middle-income countries (LMIC). Income inequality and gross domestic product (GDP) per capita has been associated to stroke mortality in developed countries. In LMIC, GDP per capita is considered to be a more relevant health determinant than income inequality. This study aims to investigate if income inequality is associated to stroke mortality in Brazil at large, but also on regional and state levels, and whether GDP per capita modulates the impact of this association.

**Methods:**

Stroke mortality rates, Gini index and GDP per capita data were pooled for the 2002 to 2009 period from public available databases. Random effects models were fitted, controlling for GDP per capita and other covariates.

**Results:**

Income inequality was independently associated to stroke mortality rates, even after controlling for GDP per capita and other covariates. GDP per capita reduced only partially the impact of income inequality on stroke mortality. A decrease in 10 points in the Gini index was associated with 18% decrease in the stroke mortality rate in Brazil.

**Conclusions:**

Income inequality was independently associated to stroke mortality in Brazil.

## Introduction

Stroke accounted for 11.1% of all deaths globally in 2010 [1], most occurring in low- and middle-income countries (LMIC) [2,3]. In recent years stroke mortality rates in Brazil decreased annually by about 4%, primarily in the affluent south [4]. Yet, due to population growth and a delayed epidemiologic transition, the number of stroke deaths actually increased [5]. Besides the recommendation to implement effective strategies targeting noncommunicable diseases (NCDs) traditional risk factors, e.g. tobacco control, salt reduction, promotion of healthy lifestyles and access to essential drugs and technology [6], socioeconomic policies to address health inequities have been advocated to tackle stroke and other NCDs [7]. Even though there is evidence of an association between low socioeconomic status (SES) and stroke in LMIC [8,9], studies analyzing the impact of income inequality—the gap between the rich and the poor—on stroke mortality in these settings are, to our knowledge, scarce.

In recent years, the relationship between income inequality and health has been well established [10–12], although in Brazil few studies have evaluated this association [13,14]. Furthermore there are methodological and theoretical controversies about the actual significance of income inequality as a health determinant [11,15,16], particularly in LMIC, since the evidence of the positive association between income inequality and ill health derives mostly from studies in high-income countries (HIC) [13,16].

Additionally, in LMIC, gross domestic product (GDP) per capita have been shown more relevant than income inequality as a determinant of health outcomes [17], which supports the notion of a wealth threshold to the income inequality effects on health [18]. Yet, the relationship of GDP per capita, economic growth and income inequality is dependent on national demographics and investments priorities [19], and in the case of Brazil, a pattern of regional differences has also been demonstrated, i.e., poorer regions have experienced increased income inequality despite substantial national economic growth [20].

The socioeconomic distribution of NCDs traditional risk factors [3,21,22] and of access to health care have been suggested as pathway mediators of the association between income inequality and stroke, though it does not fully explain disparities in stroke risk in HIC [3]. The neo-materialist model proposes that individual income and underinvestment on social infrastructure are the main explanation of the association between income inequality and health [23]. Other explanatory factors of how inequality affects health are mainly psychosocial and social capital theories. The former has described how socioeconomic disparities and the ensuing social comparison results in psychological stress detrimental to health [24]. The latter investigates the fact that egalitarian societies seem to have higher levels of trust and social participation—proxies of social capital—that are associated to better health, thus linking income distribution and health through social capital [10,25].

Brazil, which witnessed major socioeconomic improvements in the beginning of the 21st century [14,26], remains one of the most unequal countries in the world [27]. Along with the economic growth, health and social reforms have contributed to recent socioeconomic and health development [28]. The ongoing socioeconomic transition in Brazil—including income inequality levels—alongside the high burden of stroke, provide an appropriate setting to assess the relationship between income inequality, economic growth and stroke mortality, comparable to a natural experiment [29].

This study aims therefore to (i) verify the relationship between income inequality and stroke mortality and (ii) test whether and to what extent GDP per capita can reduce the impact of income inequality on stroke mortality in Brazil, and (iii) identify potential geographical regional differences in those associations.

## Methods

### Data and Measures

We analyzed publicly available data from the Brazilian Ministry of Health (Mortality Information System, the National Agency of Supplementary Health Care and the Public Health Budget Information System) and the Brazilian Institute of Geography and Statistics—IBGE (National Demographic Censuses and inter-census estimates and the National Household Sample Surveys—PNAD) [30].

The Brazilian states, including the federal district, (n = 27) was the unit of analysis. The study period was from 2002 to 2009. This time interval was due to data availability, but also socioeconomic development. A balanced longitudinal panel data set [31,32], in which each entity (i.e. states) had observations at all points in time, was assembled from the abovementioned databases. Exclusively ecological variables were included to avoid cross-level bias [33,34].

Stroke was defined according to the International Classification of Diseases (ICD-10), codes I60-I69 [30]. The dependent variable, stroke mortality rate (i.e., number of deaths per 100,000) was age standardized to the 2010 Brazilian population by the indirect method [34,35]. The Gini index indicator had been calculated based on PNAD data, using the Brown formula [30,36]. The Gini index, a frequently used indicator of income distribution, varies from 0 to 100, where a value of 0 corresponds to perfect equality and a value of 100 to complete inequality. GDP per capita data was added from IBGE, in Brazilian Reais (BRL) and its trends mark economic development. Values were not corrected for inflation and therefore do not effectively reflect the actual economic development [30]. The units of the Gini index and the GDP per capita variables were scaled based on the average change of these variables during the study period. Gini index of 55 was introduced as 5.5 and GDP per capita of 10000 BRL were introduced as 10, for example.

A set of covariates that could potentially confound the association between stroke mortality, income inequality and GDP per capita was selected based on the literature [17,37,38]: the proportion of population living in urban areas (urban population %), the proportion of health care expenditure per capita by each state (state health expenditure, %), the proportion of people living in poverty, i.e. household income below half minimum wage per capita (poverty, %), the proportion of males (male, %), mean life expectancy at birth in years (life expectancy, years), the proportion of functional illiterates, i.e. people aged 15 years old or more who cannot read or write a simple note (illiteracy, %), and the proportion of people aged 15 years old or more with 8 or more years of schooling (education ≥8years, %).

### Statistical analysis

Multivariable Poisson regression analyses for panel data modeling using random effects models were performed [39,40]. These models can be generally determined: ln(*y*_*it*_) = *β*1*x*_*it*_+*α*_*i*_+*υ*_*it*_, in which, states (_i_) were observed over time (_t_). All variables (independent, dependent and covariates) had a double subscript since they assumed a value in state_i_, at time_t_. Thus the number of observations in the regression models refers to the number of states times the number of years of follow up. The other two terms of the model account each for the unobserved time-invariant characteristics in state_i_, the individual-specific effect (α_i_) and for the idiosyncratic error term (*υ*_it_).

Poisson regression analyses are recommended for numerical data involving counting such as stroke deaths and stroke mortality rate. Negative binomial regression models were also fitted to test for the possibility of over dispersion and model fit was assessed with the Akaike's Information Criterion (AIC) and the Bayesian Information Criterion (BIC), supporting the Poisson models [39,41]. Furthermore, the statistically significant likelihood ratio test of the variance indicated the use of a panel data model, considering that there is variance across entities [41], i.e. states.

Random effects models were chosen based on the efficiency and consistency of the estimations, evidenced by the Hausman test. In addition, the random effects specification gives a more comprehensive model since it uses a weighted average of the between-states and the within-states (over time) estimations. Since the random effects models deals with both the within- and between-cluster variations combined, an additional analysis using a hybrid model—i.e. within-between approach, was fitted, providing information regarding both the within- and the between-states marginal effects separately [39,42,43].

In the descriptive statistics of the states characteristics means per geographical regions in two groups, southern—South (S), Southeast (SE) and Central west (CW) regions, and northern—North (N) and Northeast (NE) regions clusters, were calculated. To visualize how variables changed across time, means per year were presented. Means from 2002 and 2009 were compared and differences assessed by t-tests. Regression models were fitted in a stepwise approach to examine whether and to what extent GDP per capita modulated the effect of income inequality on stroke mortality. Considering regional disparities, a stratified analysis for geographical regions in groups was performed. The variable time (in years) was added to the multivariable panel data models to adjust for unmeasured time-variant variables that could have affected the observed stroke mortality time trend in all states—e.g. secular trends and changes in national level policies [32]. Cluster robust standard errors with vce (bootstraps) were used in all models [40]. The incidence rate ratio (IRR) was the measure of association adopted, adding pedagogical clarity to the results interpretation. STATA 11.2 was used for data analysis.

## Results

Table 1 presents descriptive statistics of the Brazilian states by regions, grouped in two clusters: southern and northern regions, illustrating the great regional disparities in socioeconomic, demographic and health indicators.

**Table 1:** Mean stroke mortality rate and covariates for Brazil and geographical regions clustered in two groups, Brazilian states (n = 27): 2002 to 2009.

	Brazil		S, SE, CW regions		N, NE regions	
	(N 216)		(N 88)		(N 128)	
	Mean	SD	Mean	SD	Mean	SD
Stroke mortality rate	59.88	11.68	63.45	10.18	57.42	12.05
Gini index	54.76	3.73	53.21	3.80	55.83	3.29
GDP per capita (BRL)	10625.41	7156.70	16065.66	8169.72	6885.25	2525.68
Urban population (%)	80.35	8.54	88.00	5.30	75.10	6.22
State health expenditure (%)	32.67	14.06	28.61	14.22	35.45	13.31
Poverty (%)	46.07	16.58	29.66	8.79	57.35	9.87
Male (%)	49.55	0.95	49.28	0.90	49.74	0.94
Life expectancy (years)	71.24	2.56	73.66	1.10	69.57	1.85
Illiteracy (%)	13.15	7.31	7.19	2.37	17.26	6.70
Education ≥8years (%)	47.34	9.88	53.54	7.63	43.08	8.96

S = south; SE = southeast; CW = central west; N = north; NE = northeast; SD = standard deviation.

The stroke mortality rate fluctuated over the period, with a net decrease of 7.27% from 2002 to 2009 (Table 2). Socioeconomic conditions improved: a significant decrease in income inequality, GDP per capita more than doubled, the proportion of people living in poverty decreased. There was also a significant increase in the states’ share of the health financing.

**Table 2:** Mean stroke mortality rate and covariates and overall period variation, Brazilian states (n = 27): 2002 to 2009.

	2002	2003	2004	2005	2006	2007	2008	2009	%Variation 2002–2009
Stroke mortality rate	61.33	61.47	58.86	59.28	63.77	58.56	58.88	56.87	-7.27
Gini index	56.96	55.94	55.50	54.79	54.40	54.24	52.99	53.28	-7.02*
GDP per capita (BRL)	7099.00	8141.52	9064.38	9836.37	10680.50	11863.07	13718.27	14600.20	105.67*
Urban population (%)	78.39	78.99	79.57	80.13	80.67	81.20	81.69	82.17	4.82
State health expenditure (%)	27.60	29.76	33.78	32.76	33.02	33.66	35.37	35.38	28.19*
Poverty (%)	51.95	53.61	52.22	48.78	43.87	42.65	38.47	36.99	-28.80*
Male (%)	49.57	49.56	49.52	49.50	49.53	49.56	49.58	49.57	0.01
Life expectancy (years)	69.95	70.27	70.82	71.15	71.46	71.76	72.08	72.39	3.49*
Illiteracy (%)	14.19	14.11	14.05	13.80	12.85	12.36	12.17	11.72	-17.41
Education ≥8years (%)	41.61	43.80	44.32	45.92	47.85	50.02	52.34	52.89	27.11*

* Significant difference 2009 compared to 2002 (p<0.05).

**Fig 1:**
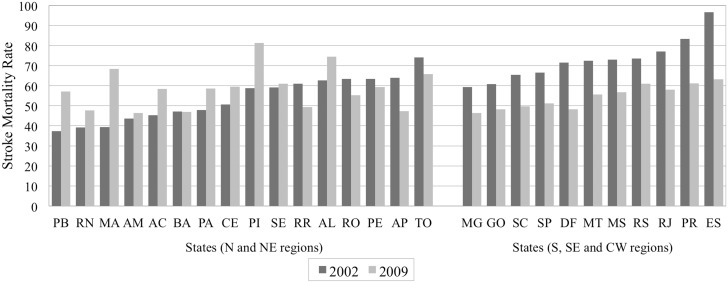
Stroke mortality rate across Brazilian states, clustered per geographical regions in two groups: 2002 and 2009.

**Fig 2:**
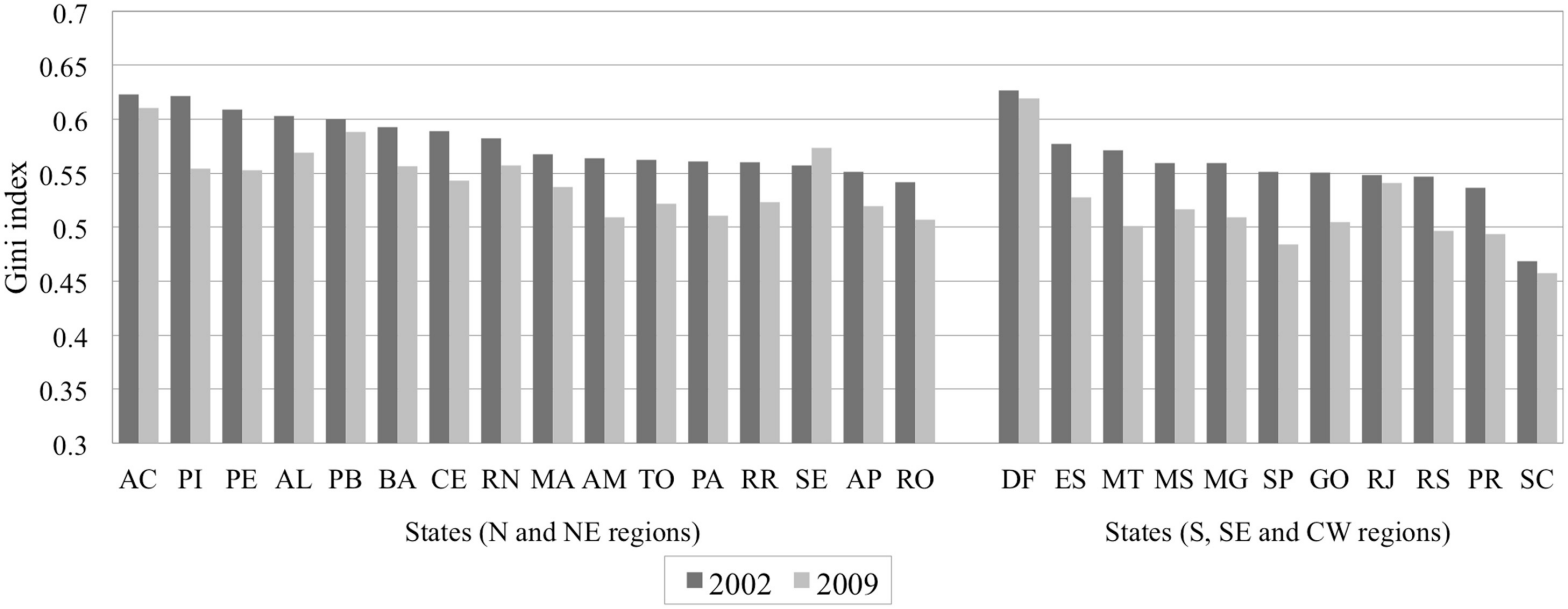
Gini index across Brazilian states, clustered per geographical regions in two groups: 2002 and 2009.

**Fig 3:**
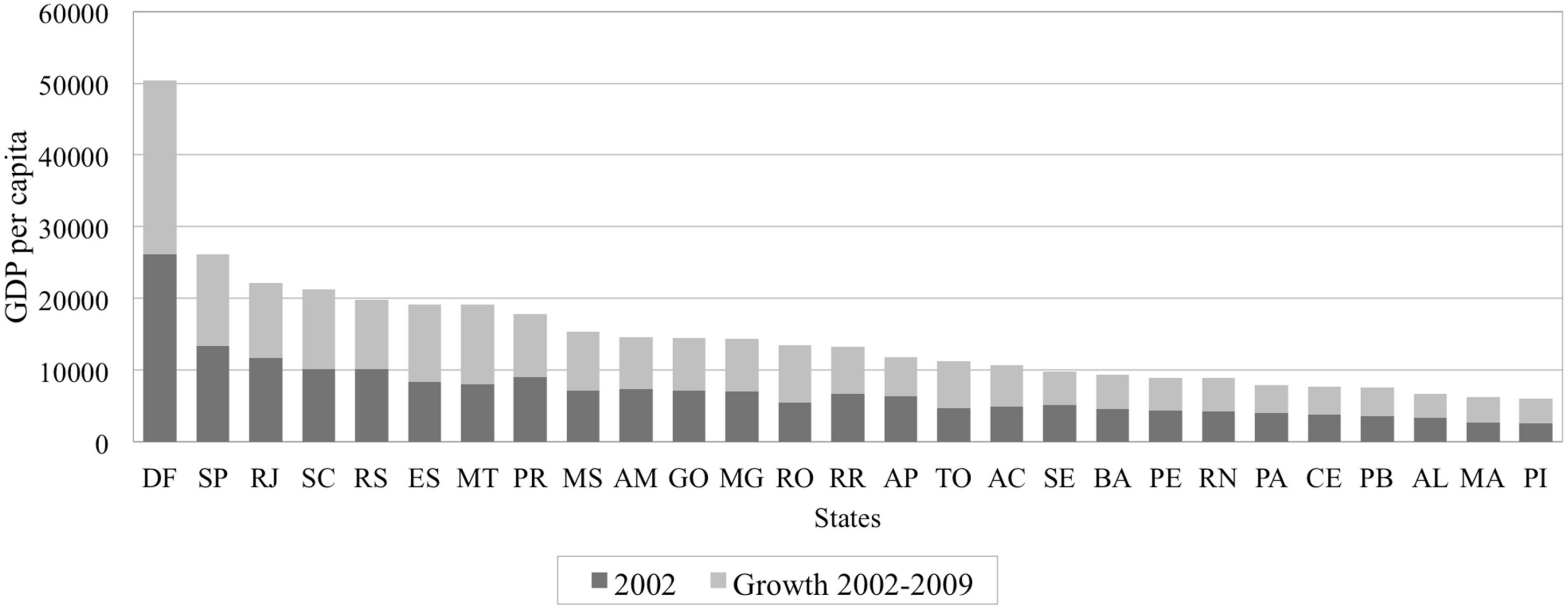
GDP per capita across Brazilian states, ranging from the highest to the lowest on 2009: values on baseline 2002 and differential from 2002 to 2009.

Within- and between-states variation of the main study variables are found in Figs 1, 2 and 3, allowing for regional distribution observations. Fig 1 presents trends of the stroke mortality rate across states, by geographical regions in two groups, using the rates of 2002 and 2009. The stroke mortality rate decreased in the states of the S, SE and CW regions but increased in most of the states in the N and NE regions. In addition, the magnitude of the variation of the mortality rates differed across states, even within geographical regions. Figs 2 and 3 illustrate the geographical distribution of Gini index and GDP per capita, respectively, showing a concentration of wealth to the southern regions.

Table 3 presents the results of the panel data model analyses. Model 1 was a bivariate analysis of stroke mortality and each of the main study variables. The higher the Gini index, the lower GDP per capita and the lower the health expenditure, the higher the stroke mortality. In model 2 we adjusted the income inequality model for GDP per capita, which was inversely associated with the stroke mortality and turned the association between Gini and stroke mortality statistically insignificant. In Model 3 we introduced several covariates, which were statistically insignificant. Yet, in Model 3 there was a positive association between income inequality and stroke mortality, independent of the GDP per capita effect on the stroke mortality.

**Table 3:** Random effects regression models of stroke mortality rates, Brazilian states (n = 27): 2002–2009.

	Model 1^a^		Model 2^b^		Model 3^b^	
	IRR	95% CI	IRR	95% CI	IRR	95% CI
Gini index	1.29	1.08–1.54	1.15	0.98–1.35	1.17	1.01–1.36
GDP per capita (BRL)	0.97	0.97–0.98	0.96	0.93–0.99	0.97	0.94–1.00
Urban population (%)	0.99	0.96–1.02			1.03	0.98–1.08
State health expenditure (%)	0.99	0.99–0.99			1.00	0.99–1.00
Poverty (%)	1.00	0.99–1,01			1.00	0.99–1.00
Life expectancy (years)	0.95	0.90–1.00			1.11	0.97–1.28
Observations	216		216		216	
Number of States	27		27		27	

IRR = incidence rate ratio; CI = confidence interval; S = south; SE = southeast; CW = central west; N = north; NE = northeast.

^a^Model 1 presents bivariate analyses.

^b^Model 2 was adjusted for time; model 3 for time, male proportion, illiteracy and education ≥8years.

The separated analysis of the within- and between-states variation effects on the stroke mortality is presented in Table 4. The within-states variation is responsible for most of the variables effects on the stroke mortality rate (evidenced by the statistical significant results and the relatively small standard errors), except for the state proportion of health expenditure. The larger the average state proportion of health expenditure in a state, the greater the stroke mortality.

**Table 4:** Within-between approach regression model of stroke mortality rates, Brazilian states (n = 27): 2002–2009.

		Model^a^		
		IRR	SE	95% CI
Within-states				
	Gini index	1.18	0.089	1.01–1.36
	GDP per capita (BRL)	0.98	0.016	0.95–1.01
	Urban population (%)	1.04	0.027	0.99–1.10
	State health expenditure (%)	1.00	0.003	0.99–1.00
	Poverty (%)	0.99	0.004	0.99–1.00
	Life expectancy (years)	1.27	0.108	1.07–1.50
Between-states				
	Gini index	3.61	6.394	0.11–116.24
	GDP per capita (BRL)	0.92	0.116	0.72–1.18
	Urban population (%)	1.01	0.043	0.93–1.10
	State health expenditure (%)	1.07	0.033	1.01–1.14
	Poverty (%)	0.92	0.053	0.82–1.03
	Life expectancy (years)	0.82	0.153	0.57–1.18
	Observations	216		
	Number of States	27		

IRR = incidence rate ratio; CI = confidence interval; SE = standard error.

^a^Model was adjusted for time, male proportion, illiteracy and education ≥8years.

We conducted a stratified analysis of the multivariable model by geographical regions in two groups: S, SE and CW states, and N and NE states. Even though the association between income inequality and stroke mortality was not statistically significant in any strata (IRR = 1.02, 95% CI = 0.87–1.18 and IRR = 1.21, 95% CI = 0.99–1.48, respectively), and neither was the GDP per capita one (IRR = 1.02, 95% CI = 0.87–1.18 and IRR = 1.21, 95% CI = 0.99–1.48, respectively), the analysis suggested relevant differences between the two clusters. Health care expenditure was negatively associated to stroke mortality only in the N and NE regions (IRR = 0.99, 95% CI = 0.98–0.99). Poverty significantly affected stroke mortality in the two groups, positively in the S, SE and CW states (IRR = 1.01, 95% CI = 1.00–1.01) but negatively in the other group (IRR = 0.99, 95% CI = 0.98–0.99). There was a positive association between life expectancy and stroke mortality in the N and NE states (IRR = 1.35, 95% CI = 1.14–1.60).

## Discussion

This study found that income inequality was independently significantly associated with higher stroke mortality in Brazil, even after adjusting for GDP per capita, urban population, health care expenditure, poverty levels and other covariates. Our analysis suggested that a decrease in 10 points in the Gini index was associated with 18% decrease in the stroke mortality rate.

These findings challenge the notion that GDP per capita, rather than income distribution, is primarily associated to health in LMIC [17]. Based on our findings, GDP per capita was at first sight, in the crude analysis, more relevant to stroke mortality than income inequality. However after considering other contextual factors, such as socioeconomics, demographics and health care financing, only income inequality was independently significantly associated to the outcome.

A recent Brazilian study applying a similar design and analytical approach found similar results regarding life expectancy although regional disparities were not explored [14]. Our stratified analysis, which could not refute the association between stroke mortality and income inequality in the northern poorer regions of the country, does not support the notion that income inequality should be of lesser importance as a health determinant in less affluent settings. Still, further studies with for instance a longer follow up and therefore a bigger number of panel data observations would be necessary to confirm these results.

Thus, since economic growth is not in itself enough to reduce health and socioeconomic disparities [19,20], policies targeting specifically income inequality in LMIC could further reduce the stroke mortality where its burden is higher. In Brazil, the recent improvements in inequality indicators were mainly attributed to a decrease in poverty levels [28] while we would claim that, which is empirically supported by our findings, a more profound general income redistribution policy is necessary [14].

Health care expenditure, although not a direct measure of health care access and quality, was considered an adequate macroeconomic proxy of stroke primary prevention and acute care based on previous research [38]. The Brazilian public health system, which is financed through federal, state and municipal tax revenues, is chronically underfunded. However in recent years the states’ share of health financing has increased, balancing the proportional decrease in federal contributions [26,44]. Our study found that the states’ share of the health care financing had over time a negative impact on the stroke mortality in the poorer northern and northeastern states. This indicates that there is an imminent effect of health care in managing the disease markedly in the states with an increasing burden of the disease. Additionally, the average health care expenditure by each state was positively associated with a greater stroke mortality rate, meaning that the state, which proportionally contributed more to the health care financing, had higher stroke mortality rates. Ultimately, since the state proportion of health expenditure mirrors the federal proportion of health expenditure, this reveals a poor national health care resources allocation policy.

It has previously been claimed that absolute income, and then especially poverty levels, could possibly single-handedly explain the association between income inequality and health. This could be explained by the “convexity effect” in which the effect of income deprivation is greater than of the income wealth on health [15,45,46]. Yet, in highly unequal settings, like Brazil, the influence of poverty on the association has been previously described as marginal [46]. In fact, in our main analysis the poverty effect on the association between income inequality and stroke mortality did not support the “convexity effect”. Moreover, in our stratified analysis the effect of poverty on stroke mortality went in two different directions depending on the geographical region: positively in the S, SE and CW and negatively in the N and NE states. This possibly reflects the relative meaning of the income threshold that defined poverty within the dataset, and it could also demonstrate how Brazilian regions are in different stages of the demographic and epidemiological transition [5].

Although the southern regions have a higher life expectancy, this indicator was positively associated with the stroke mortality rates only in the northern regions of the country. The effect therefore goes beyond population age in itself. This illustrates the abovementioned different stages of the demographic and epidemiological transition between the northern and southern regions. Focusing only on the NCDs, the epidemiologic transition from cardiovascular diseases (i.e. from rheumatic heart disease to hemorrhagic stroke to ischemic stroke to coronary heart disease) [47] to other degenerative diseases could explain the higher impact of the recently growing life expectancy in the N and NE states when compared to the advanced demographic transition in the southern regions.

The association between income inequality and stroke mortality was shown to be robust to the study period time-specific effects. However the association between income inequality and health has been previously described as dependent on the study period societal characteristics and other time bound factors [48]. Therefore, since the analysis couldn’t account for all the determinants implied by time, our results may not be easily generalized to other time periods. Yet, a multivariable model adjusted for an interaction term between income inequality and time [48] indicated that as time passed, the association between income inequality and the stroke mortality was greater, especially in the northern regions of the country (data not shown).

In Brazil, parallel studies have previously explained the impact of income inequality on health by psychosocial theories [14], and although some authors have associated social capital to health outcomes disparities in Brazil [49,50], empirical studies targeting social capital, income inequality and health are scarce. In fact, most of the social capital studies have focused on the association between low SES, social capital access/utilization and health outcomes [25], lacking the needed analysis of the contextual effect of income distribution to social capital and to health, which could further explain how income inequality affects the overall population health. Furthermore, there are conceptual and methodological issues regarding social capital studies in general [51], and therefore more studies in the area are required.

### Methodological considerations

The increasing stroke mortality rates observed in most of the states of the N and NE regions in the study period could possibly be associated with the recent improvements in data collection in those regions—e.g. reduction of poorly defined diagnoses [52,53]. However, we have not observed any strong systematic tendencies that could annul the impact of the country’s socioeconomic changes in the study period. Death certificates were the main source used to assess stroke mortality rates and although there is critique regarding its use in stroke mortality studies [54], death reports are compulsory in Brazil, which allows comprehensive analysis of death rates on population level [52].

Another study limitation is the potential of ecological fallacy, reflecting the impossibility to establish conclusions for individuals [34]. The design of the study and the research question in itself minimize the relevance of the ecological fallacy, since the ultimate goal was to establish how context affects the population. Additionally, income inequality is intrinsically an aggregate measure, and to avoid the cross level bias, no individual level data was used [33].

Heterogeneity could be a limitation of studies about income inequality and health [14,16]. However, the application of panel data analysis, allowed for the adjustment of states’ heterogeneity in the time invariant characteristics [31], such as stable state level policies and historical and cultural aspects. Even though it was expected that the unobserved time-invariant states characteristics abovementioned were correlated with the studied regressors, the random effects modeling assumed otherwise, which could theoretically lead to omitted variable bias. However, because of a significantly smaller within- rather than between-cluster standard errors, the random effects models estimations were weighted towards the within-cluster estimations, determining unbiased results very similar to the fixed-effects models, yet more comprehensive [42,43]. Thus, the use of the within-between approach, which allowed both the over time and between-states processes to be explicitly modeled, is considered another study strength.

## Conclusion

Income inequality trends were independently associated with stroke mortality rate trends in Brazil, even after adjusting for economic growth and other covariates. These findings support the notion that income inequality is a relevant health determinant in LMIC and further emphasize the role of income inequality, besides wealth, in relation to health outcomes. The association between income inequality and stroke mortality has several policy implications in LMIC, like Brazil, from directly targeting income distribution to tackling other social determinants of health with a territorial approach, aiming at reducing health inequities. To confirm the regional particularities of the income inequality and stroke mortality association further studies are necessary.
